# Automatic detection of abnormal hand gestures in patients with radial, ulnar, or median nerve injury using hand pose estimation

**DOI:** 10.3389/fneur.2022.1052505

**Published:** 2022-12-07

**Authors:** Fanbin Gu, Jingyuan Fan, Chengfeng Cai, Zhaoyang Wang, Xiaolin Liu, Jiantao Yang, Qingtang Zhu

**Affiliations:** ^1^Department of Microsurgery, Orthopedic Trauma and Hand Surgery, The First Affiliated Hospital, Sun Yat-sen University, Guangzhou, China; ^2^Department of Hand and Foot Rehabilitation, Guangdong Provincial Work Injury Rehabilitation Hospital, Guangzhou, China; ^3^Guangdong Provincial Engineering Laboratory for Soft Tissue Biofabrication, Guangzhou, China; ^4^Guangdong Provincial Key Laboratory for Orthopedics and Traumatology, Guangzhou, China

**Keywords:** peripheral nerve injury, hand pose estimation, hand gesture, abnormal gesture detection, expert system, machine learning

## Abstract

**Background:**

Radial, ulnar, or median nerve injuries are common peripheral nerve injuries. They usually present specific abnormal signs on the hands as evidence for hand surgeons to diagnose. However, without specialized knowledge, it is difficult for primary healthcare providers to recognize the clinical meaning and the potential nerve injuries through the abnormalities, often leading to misdiagnosis. Developing technologies for automatically detecting abnormal hand gestures would assist general medical service practitioners with an early diagnosis and treatment.

**Methods:**

Based on expert experience, we selected three hand gestures with predetermined features and rules as three independent binary classification tasks for abnormal gesture detection. Images from patients with unilateral radial, ulnar, or median nerve injuries and healthy volunteers were obtained using a smartphone. The landmark coordinates were extracted using Google MediaPipe Hands to calculate the features. The receiver operating characteristic curve was employed for feature selection. We compared the performance of rule-based models with logistic regression, support vector machine and of random forest machine learning models by evaluating the accuracy, sensitivity, and specificity.

**Results:**

The study included 1,344 images, twenty-two patients, and thirty-four volunteers. In rule-based models, eight features were finally selected. The accuracy, sensitivity, and specificity were (1) 98.2, 91.7, and 99.0% for radial nerve injury detection; (2) 97.3, 83.3, and 99.0% for ulnar nerve injury detection; and (3) 96.4, 87.5, and 97.1% for median nerve injury detection, respectively. All machine learning models had accuracy above 95% and sensitivity ranging from 37.5 to 100%.

**Conclusion:**

Our study provides a helpful tool for detecting abnormal gestures in radial, ulnar, or median nerve injuries with satisfying accuracy, sensitivity, and specificity. It confirms that hand pose estimation could automatically analyze and detect the abnormalities from images of these patients. It has the potential to be a simple and convenient screening method for primary healthcare and telemedicine application.

## Introduction

Peripheral nerve injury (PNI) is devastating and frequently results in life-long disability, severely decreasing patients' quality of life. It affects more than one million people worldwide, with an incidence of ~13–23 per 100,000 persons per year (1, 2). Among PNIs of the upper limb, radial, ulnar, and median nerve injuries are common, causing sensory and motor dysfunction in the control area of the damaged nerves. In medical practice, early detection of nerve injury may prevent patients from delayed diagnoses, delayed repairs, and potentially worse outcomes. However, the anatomical complexity of the hand may bring difficulties in examination and diagnosis (3). According to a large German database of hand and forearm injuries, nerve injuries were among the most commonly missed injuries, accounting for 24.8% of missed hand injuries (4). The misdiagnosis rate would be considerably higher in areas with insufficient specialized services. In China, for example, there have been reports that most treated patients with missed nerve injuries were from primary healthcare hospitals (5, 6).

The clinical diagnosis of nerve injury depends on clinical history, clinical symptoms, and physical and neurological examinations (7, 8). Among the examinations, electromyography (EMG), nerve conduction studies (NCS), MRI, and high-resolution ultrasonography have been successfully used as diagnostic methods for PNI (9–11). However, these examinations are expensive, invasive, and rely on specialized equipment and professional operators, restricting their application in primary assessment. Given these problems, there is a need for an affordable, non-invasive, and accessible method to detect possible nerve injuries for initial evaluation of the hand, significantly helping clinicians who are not specialized in hand surgery and benefiting patients in remote or rural areas. Some abnormal signs or deformities of the hand may indicate the nerve injuries, such as the limited extension of the wrist and digits for radial nerve injury, the claw hand deformity and the Wartenberg sign for ulnar nerve injury, and the ape hand deformity for median nerve injury. These clinical experiences have been well-acknowledged in hand surgery, where nerve injuries can lead to various morphological changes based on the different innervation of the hand (12, 13). So far, deformity of digit(s)' joint(s) has been used to describe specific nerve injury as supporting evidence (14–16). This unique connection between abnormalities and nerve injury was determined by the independent anatomical nerve innervation to the muscle. Additionally, it is possible to observe these abnormalities through several gestures. Therefore, detecting abnormal gestures can be a simple method for predicting hand nerve injuries.

With the advancement in computer vision, hand pose estimation is possible using automatic analysis techniques for detecting the hand and predicting the articulated joint locations from images or videos (17, 18). Hand pose estimation is crucial and popular in achieving several tasks, such as gesture recognition (19), action recognition (20), and sign language recognition (21), in general populations. There has been an increasing interest in hand pose estimation techniques for medical research and application, such as investigating automatic assessment of hand rehabilitation (22) and using 3D cameras for the classification of Parkinson's disease (23). These techniques seem to help extract the features of the hand. Among them, MediaPipe Hands is a deep-learning-based hand-tracking solution provided by Google. It does not require specialized hardware and is adequately light to run in real-time on mobile devices; it can also predict 21 landmarks of a hand from a single RGB camera with high prediction quality (24). MediaPipe Hands are popular in human-computer interaction systems and applications of virtual and augmented reality (21, 24, 25). However, it remains unknown whether MediaPipe Hands could detect the abnormalities in patients with nerve injuries, distinguish normal and abnormal gestures in the medical application, and further predict possible injuries based on rule-based methods.

The purpose of the present study was to propose an automatic method for abnormal hand gesture detection caused by radial, ulnar, or median nerve injury. We hypothesize that hand pose estimation could provide specific features to classify the presence of nerve injury and predict the exact type of injury.

## Materials and methods

### Participants recruitment

**Figure 1:**
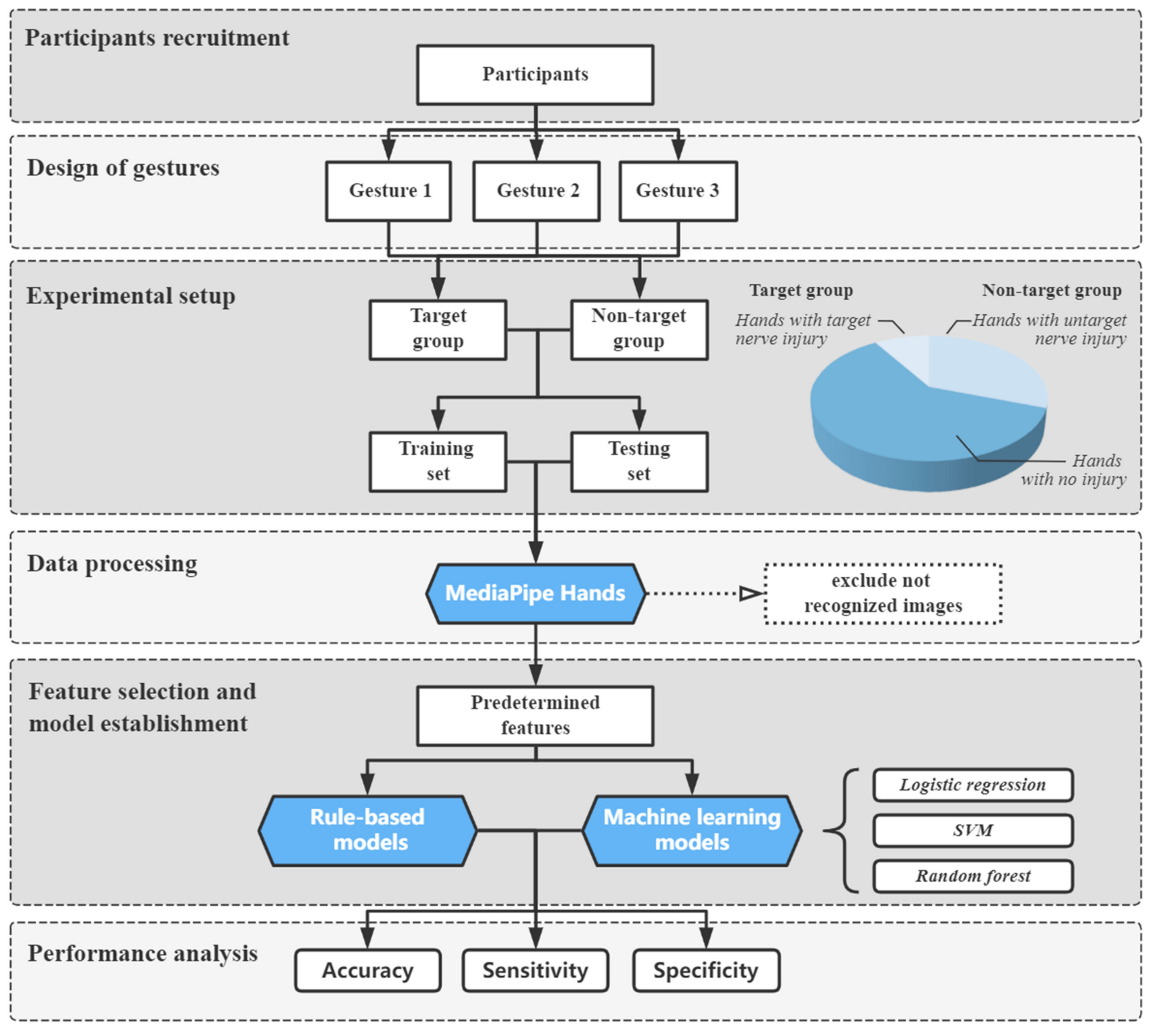
Diagram of the experimental procedure.

The experimental procedure of this study is illustrated in Figure 1. From June 2021 to October 2022, we recruited preoperative patients with unilateral radial, ulnar, or median nerve injuries who were scheduled to undergo surgery in our department. The diagnosis was made after a comprehensive evaluation of the related history, the observation of typical abnormal gestures, and EMG or ultrasound examination, which was further confirmed in the surgical exploration. Patients with any musculoskeletal disease (such as tendon rupture or arthritis) and neurological diseases (such as stroke and traumatic brain injury) that would influence the movement of the hands (not including the wrist joint) and those who refused to participate were excluded. A group of healthy volunteers were also invited during this period. The inclusion criteria for the volunteers were the absence of symptoms such as clumsiness or numbness of hands and no abnormal findings in the physical examination that indicated nerve injuries. All participants understood the study's protocol and cooperated in taking the images. Verbal consent was obtained for the usage of their images for research purposes. This study was approved by the institutional review board of the First Affiliated Hospital of Sun Yat-sen University (ID: [2021]387).

### Design of gestures

**Figure 2:**
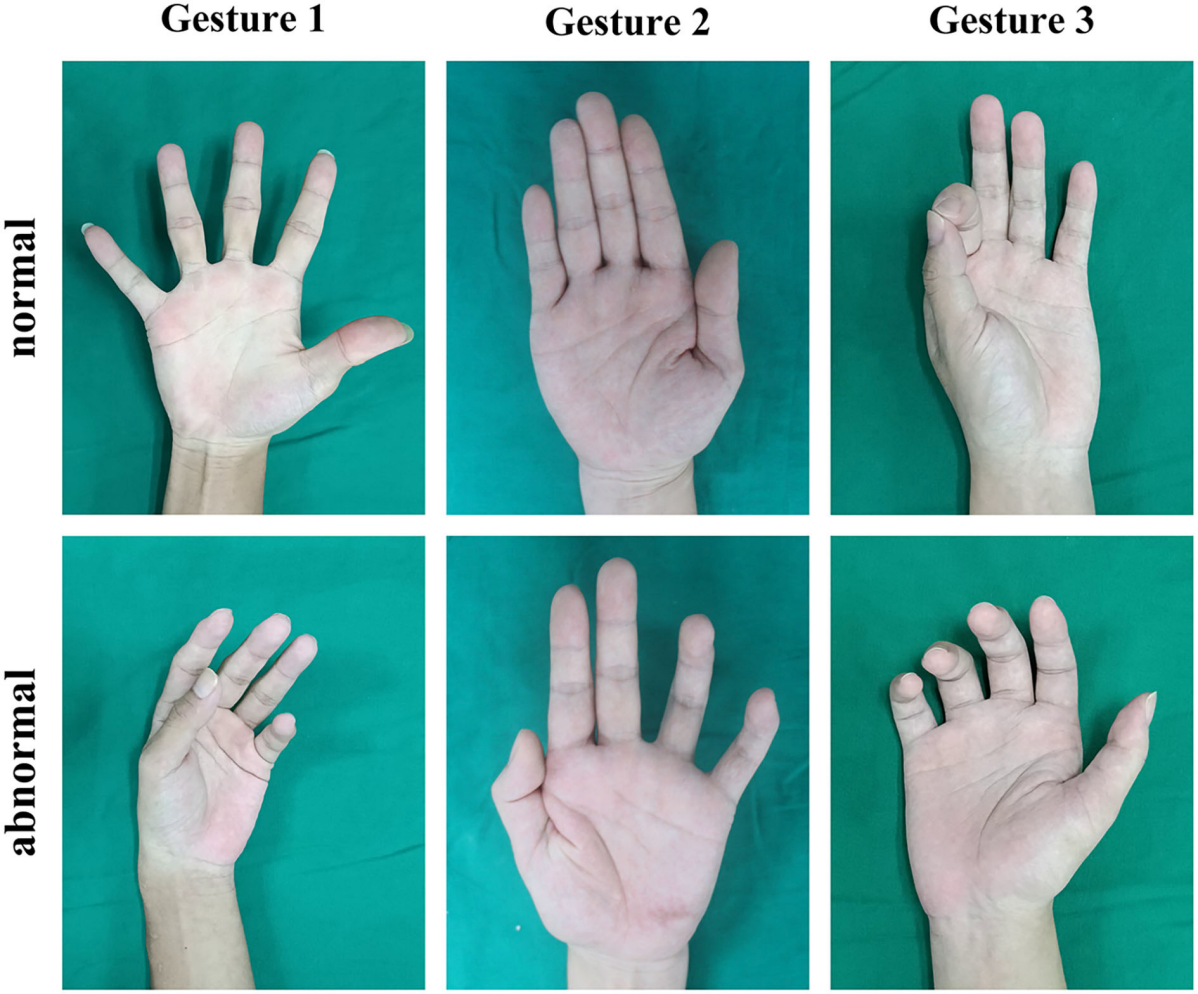
Samples of normal and abnormal gestures.

Based on the expert experience mentioned above (12, 13), we chose three gestures (Figure 2) to detect abnormalities caused by radial, ulnar, or median nerve injuries as three binary classification tasks. The normal and the expected abnormal gestures were demonstrated as follows:

- Gesture 1 was used to detect radial nerve injuries. Participants were requested to make a maximum abduction of digits to the medial and lateral sides with full extension. Patients with radial nerve injury would have impaired functions of the musculus abductor pollicis longus, the musculus extensor pollicis longus, the musculus extensor pollicis brevis, and the musculus extensor digitorum. The patients were expected to have difficulties extending all metacarpophalangeal (MCP) joints.
- Gesture 2 was used to detect ulnar nerve injuries. Participants were requested to make adduction of digits in full extension toward the middle finger. Patients with ulnar nerve injury would have impaired the function of the musculus lumbricales 3 and 4 and the musculus interosseous palmaris. This would lead to flexed proximal interphalangeal (PIP) joints and distal interphalangeal (DIP) joints of the ring and little fingers (the claw hand deformity) and limited adduction of the ring and particularly the little fingers (the Wartenberg sign).
- Gesture 3 was used to detect median nerve injuries. Participants were requested to perform a tip-to-tip pinch between the thumb and the index finger, i.e., to form an “OK” gesture. Patients with median nerve injury would have impaired functions of the musculus opponens pollicis, the musculus flexor pollicis brevis, and the musculus lumbricales 1 and 2. When performing this gesture, the decreased range of palmar abduction of the thumb would occur, and it could have trouble flexing the thumb and index finger, even a failure to make contact with the tips of the two digits.

### Experimental setup

Before taking images, all participants washed and cleaned their hands. Jewelry, watches, and clothing on the wrist or hand were removed. Then, the hand was placed on the test table with the forearm kept in a supination position. Participants would first perform Gesture 1 for radial nerve classification. Injured hands with radial nerve injury were put in the target group; injured hands with ulnar or median nerve injury and hands without nerve injury were put in the non-target group. Then, stratified random sampling was used between the target and non-target groups to form the training and testing sets. The ratio of training to testing sets was ~3:1. Next, Gesture 2 and Gesture 3 were performed in the same way to separate respective training and testing sets.

Each gesture was requested to be performed four times with a rest interval of ~10 s to obtain more available data. For every gesture of a hand, four images were obtained. The images were taken 40–50 cm above the hand between a slightly radial view and a slightly ulnar view using a smartphone (iPhone XS Max, Apple Inc., image resolution: 1,980^*^1,080 p) to make them more different. If the forearm had limited supination, images would be taken from the exact distance parallel to the palm.

### Data processing

**Figure 3:**
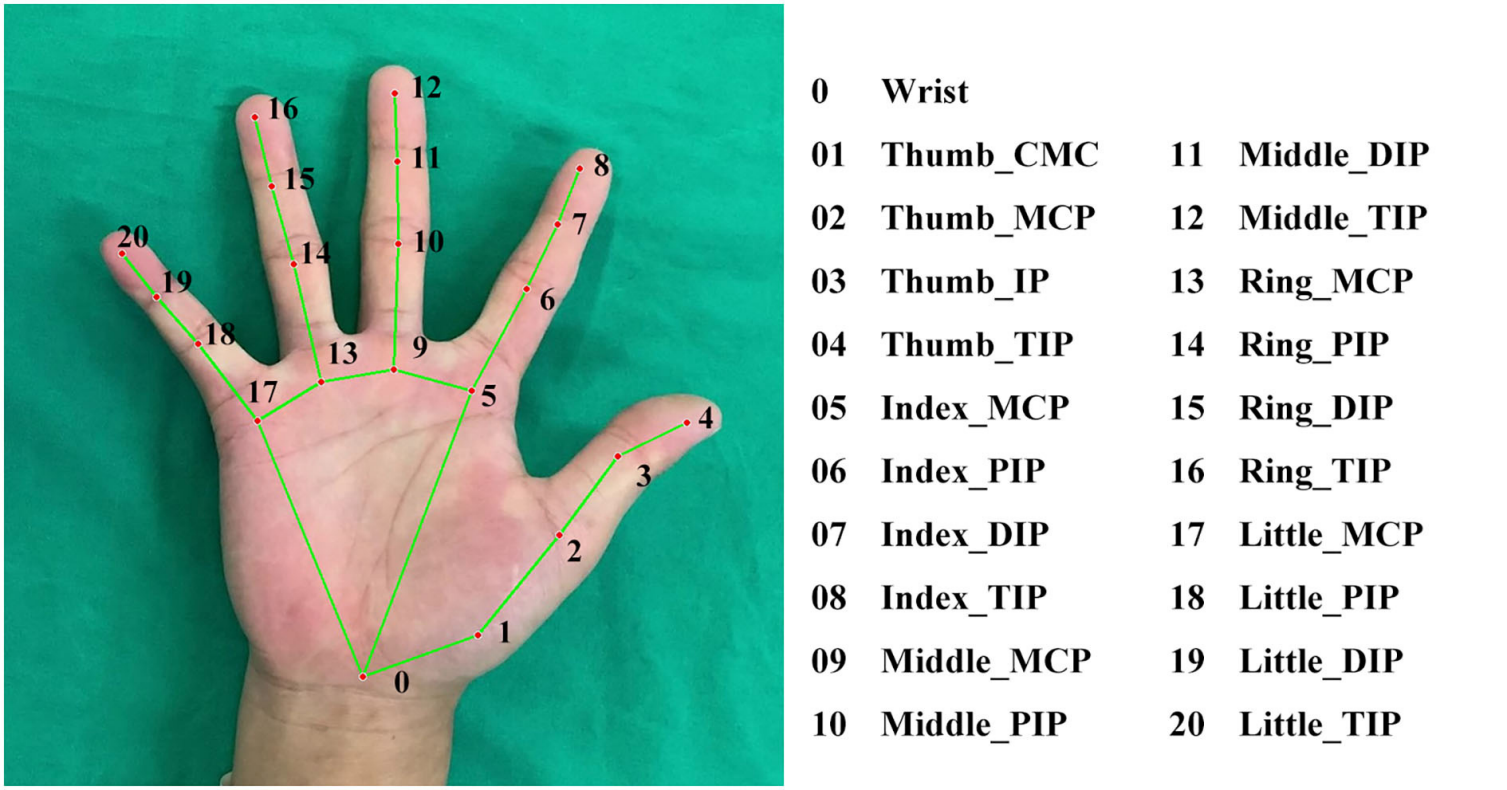
The 21 landmarks of the skeleton model of MediaPipe Hands.

All images were analyzed using MediaPipe Hands solution (Version 0.8.9) with Python (Version 3.8). MediaPipe Hands would first detect and locate the hand through a palm detector algorithm. If the hand was successfully located in the image, then the coordinates of the landmarks could be obtained by its landmark model (24) (Figure 3). If the algorithm did not detect the hand, the image could not be recognized and was excluded from further analysis. After receiving the coordinates, features could be automatically calculated using the following equations:


(1) $$\begin{array}{l} \theta ={\text{cos}}^{-1}\left(\frac{\vec{S_{1}}\cdot \vec{S_{2}}}{\left|\vec{S_{1}}|\cdot |\vec{S_{2}}\right|}\right) \end{array}$$

(2) $$\begin{array}{l} d=\sqrt{{\left(x_{1}-x_{2}\right)}^{2}+{\left(y_{1}-y_{2}\right)}^{2}+{\left(z_{1}-z_{2}\right)}^{2}} \end{array}$$

(3) $$\begin{array}{l} d^{\prime }=\frac{d_{original}}{d_{standard}} \end{array}$$

where

Equation (1) calculates the targeted angle θ, where $\vec{S_{1}}$ and $\vec{S_{2}}$ represent the vectors of the related phalanges (26);

Equation (2) calculates the targeted distance *d*, where *x*_1_, *y*_1_, *z*_1_ and *x*_2_, *y*_2_, *z*_2_ represent the coordinates of the related landmarks;

Equation (3) standardizes any distance values *d*′ by dividing its original distance *d*_*original*_ by the distance between the landmarks of the tip and IP joints of the thumb *d*_*standard*_.

### Feature selection and model establishment

Since the radial, ulnar, and median nerves have independent innervations to the muscle (mentioned in the “Design of gestures” section), medical knowledge and expert experience can provide a series of primary and anatomical-based features. For example, the ulnar nerve is related to the movement of the ring and little fingers, so the features of these two fingers are much more important than the other digits, and irrelevant features could be ignored. In this consideration, we predetermined 23 features with our experience, including joints' angles, key point's distance, and their combinations (Table 1), for feature selection and model establishment.

**Table 1:** Description and statistical analysis of the predetermined features.

**No**.	**Feature name**	**Description**	**Group**	**Mean**	**SD**	**95% CI**	***P***	**AUC**
**Gesture 1**
01	**ag_thumb_MCP**	Angle of thumb MCP joint	Non-target	5.49	3.59	(5.083, 5.889)	0.00	0.95
			Target	21.36	7.27	(18.540, 24.181)		
02	**ag_index_MCP**	Angle of index MCP joint	Non-target	8.46	3.30	(8.090, 8.831)	0.00	0.85
			Target	19.02	9.95	(15.163, 22.876)		
03	**ag_middle_MCP**	Angle of middle MCP joint	Non-target	4.87	2.39	(4.606, 5.142)	0.00	0.89
			Target	17.44	11.74	(12.884, 21.987)		
04	**ag_ring_MCP**	Angle of ring MCP joint	Non-target	6.99	3.14	(6.639, 7.343)	0.00	0.85
			Target	19.80	11.87	(15.203, 24.407)		
05	ag_little_MCP	Angle of little MCP joint	Non-target	13.75	6.37	(13.038, 14.466)	0.00	0.68
			Target	24.73	16.13	(18.479, 30.989)		
**Gesture 2**
06	ag_ring_PIP	Angle of ring PIP joint	Non-target	6.75	7.08	(5.950, 7.548)	0.00	0.68
			Target	13.81	12.95	(9.145, 18.482)		
07	ag_ring_DIP	Angle of ring DIP joint	Non-target	4.74	6.28	(4.030, 5.447)	0.00	0.68
			Target	13.05	15.13	(7.596, 18.507)		
08	ag_ring	Sum of feature 06 and 07	Non-target	11.49	12.51	(10.076, 12.899)	0.00	0.67
			Target	26.87	27.13	(17.084, 36.646)		
09	ag_little_PIP	Angle of little PIP joint	Non-target	7.02	4.48	(6.511, 7.523)	0.00	0.66
			Target	12.46	9.35	(9.086, 15.828)		
10	ag_little_DIP	Angle of little DIP joint	Non-target	5.85	5.72	(5.208, 6.498)	0.00	0.71
			Target	13.70	12.43	(9.218, 18.184)		
11	ag_little	Sum of feature 09 and 10	Non-target	12.87	9.57	(11.790, 13.950)	0.00	0.66
			Target	26.16	21.39	(18.445, 33.871)		
12	**web3**	Angle of the third webspace	Non-Target	1.58	1.00	(1.462, 1.688)	0.00	0.99
			Target	6.50	1.83	(5.837, 7.158)		
13	**web4**	Angle of the fourth webspace	Non-target	1.75	1.51	(1.583, 1.924)	0.00	0.89
			Target	8.33	5.27	(6.433, 10.234)		
**Gesture 3**
14	ag_thumb_CMC*	Angle of thumb CMC joint	Non-target	−28.16	6.62	(−28.902, −27.427)	0.19	0.59
			Target	−30.16	5.61	(−32.785, −27.534)		
15	ag_thumb_MCP*	Angle of thumb MCP joint	Non-target	−24.10	9.88	(−25.198, −22.997)	0.01	0.65
			Target	−30.61	12.01	(−36.229, −24.991)		
16	ag_thumb_IP*	Angle of thumb IP joint	Non-target	−42.20	19.62	(−44.39, −40.019)	0.72	0.49
			Target	−39.89	28.04	(−53.009, −26.765)		
17	ag_thumb*	Sum of feature 14, 15, and 16	Non-target	−94.47	19.51	(−96.64, −92.293)	0.38	0.61
			Target	−100.66	30.51	(−114.934, −86.378)		
18	ag_index_MCP*	Angle of index MCP joint	Non-target	−32.72	5.41	(−33.327, −32.122)	0.03	0.39
			Target	−29.91	6.87	(−33.129, −26.694)		
19	ag_index_PIP*	Angle of index PIP joint	Non-target	−70.28	18.33	(−72.319, −68.237)	0.02	0.33
			Target	−55.08	25.78	(−67.146, −43.018)		
20	ag_index_DIP*	Angle of thumb DIP joint	Non-target	−36.99	13.85	(−38.531, −35.445)	0.62	0.54
			Target	−40.21	28.77	(−53.675, −26.748)		
21	ag_index*	Sum of feature 18, 19, and 20	Non-target	−139.99	19.53	(−142.166, −137.815)	0.20	0.60
			Target	−125.21	49.30	(−148.279, −102.131)		
22	ag_palmab*	Angle of thumb palmar abdution	Non-target	−19.95	8.34	(−20.881, −19.023)	0.00	0.87
			Target	−9.30	4.43	(−11.379, −7.231)		
23	**dis_tip**	Distance of thumb tip to index tip	Non-target	0.72	0.28	(0.693, 0.756)	0.02	0.66
			Target	1.19	0.81	(0.813, 1.575)		

SD, standard deviation; CI, confidence interval; AUC, area under the curve. The unit for all angle features was degree. Feature 23 was a standardized value with no unit. Features after selection in the rule-based models were presented in bold.

*Represents a negative value that was taken for this feature to adapt the classification rules.

In the study, two different methods for model establishment were compared. One was the rule-based method, which means that the model was built under a manually designed feature selection and decision-making processes based on knowledge and experience. The other was the machine learning (ML) method, which means that the whole process was learned from the data. The logistic regression (LR) model, the support vector machine (SVM) model, and the random forest (RF) model were chosen for the ML method. For each gesture, one rule-based model and three ML models were established.

In the rule-based method, feature selection for the predetermined features was analyzed using the receiver operating characteristic (ROC) curve, a standard method to assess the performance of binary classification models (27, 28). The classification efficacy of features could be assessed by calculating the area under the curve (AUC), which was thought to be poor (< 0.6), fair (0.6–0.7), good (0.7–0.8), very good (0.8–0.9), or excellent (>0.90) (29). We calculated the AUC of predetermined features using the training sets, filtered features with an AUC below 0.8, and selected at least two features in each gesture classification model as the classifiers. Next, we determined the threshold value of each classifier according to ROC curves. Finally, all classifiers were ensembled according to the rules of the model: if every classifier was below the threshold, the gesture was predicted as normal and regarded as uninjured; otherwise, the gesture was predicted as abnormal and regarded as injured.

In the ML models, all predetermined features were taken, standardized, and trained in the training sets using scikit-learn python application program interface (API) (https://scikit-learn.org.cn/). Grid search with 5-fold cross-validation was performed to find the best hyperparameters, enhancing the efficacy of the ML models. The feature selection of ML models was “embedded” and completed in the training process. We used “coef_” API in the LR and SVM models and “feature_importances_” API in the RF model to evaluate the importance of features and compare the results of feature selection between rule-based and machine learning methods.

### Performance analysis

The performance of the rule-based model and the LR, SVM, and RF models was evaluated using the testing sets for accuracy, sensitivity, and specificity. The confusion matrixes were used to visualize the agreement between the prediction and the actual label. The indices used in this study were calculated as follows:


$$\begin{array}{l} accuracy=\;\frac{TP+TN}{TP+FP+TN+FN}\times 100\%\;\\ sensitivity=\;\frac{TP}{TP+FN}\;\times 100\%\;\\ specificity=\;\frac{TN}{FP+TN}\times 100\%\; \end{array}$$

where

TP represents the number of abnormal gestures correctly predicted as abnormal,

FP represents the number of normal gestures wrongly predicted as abnormal,

FN represents the number of abnormal gestures wrongly predicted as normal,

TN represents the number of normal gestures correctly predicted as normal.

### Statistical analysis

For predetermined features, the mean values in all target and non-target groups were presented as mean ± standard deviation (SD) with a 95% confidence interval (CI). An independent *t*-test was used to analyze the difference between the target and non-target groups. Subgroup analysis was also performed for features in each rule-based model with an independent *t*-test. A *P*-value of < 0.05 was considered statistically significant. The statistical analysis was performed using IBM SPSS version 25 (IBM, Armonk, NY, USA).

## Results

The study included twenty-two patients and thirty-four healthy volunteers who met the inclusion and exclusion criteria. The demographic of the participants is summarized in Table 2, where the mean ages of patients and volunteers were 33.04 ± 12.00 and 34.67 ± 13.84, respectively. Detailed information on patients is shown in Table 3. Among the patients, ten were diagnosed with radial nerve injury, five with ulnar nerve injury, one with median nerve injury, and six with combined ulnar and median nerve injuries. We obtained 1,344 images in total, with 448 images in each gesture classification task. MediaPipe Hands failed to recognize four images, and the overall recognition rate was 99.7%. All non-recognized images belonged to the training set of Gesture 3. In total, three rule-based models and nine ML models were established.

**Table 2:** Demographics of the participants.

	**Patients (*N* = 22)**	**Volunteers (*N* = 34)**
Age (year)	33.04 ± 12.00	34.67 ± 13.84
Gender (male)	21 (95.5%)	17 (50%)
Dominate hand (right)	22 (100%)	34 (100%)

**Table 3:** Characteristics of the patients.

**No**.	**Gender**	**Age**	**Injured side**	**Radial nerve injury**	**Ulnar nerve injury**	**Median nerve injury**
P1	Men	67	Right		√	√
P2	Men	27	Left		√	√
P3	Men	36	Left	√		
P4	Women	27	Left	√		
P5	Men	36	Right	√		
P6	Men	37	Right		√	
P7	Men	31	Left		√	
P8	Men	29	Right		√	
P9	Men	58	Right	√		
P10	Men	26	Left	√		
P11	Men	19	Right		√	√
P12	Men	21	Right		√	
P13	Men	26	Right			√
P14	Men	21	Left	√		
P15	Men	32	Left	√		
P16	Men	35	Left	√		
P17	Men	36	Right	√		
P18	Men	27	Left		√	√
P19	Men	17	Right		√	√
P20	Men	32	Left	√		
P21	Men	46	Left		√	√
P22	Men	41	Right		√	

**Figure 4:**
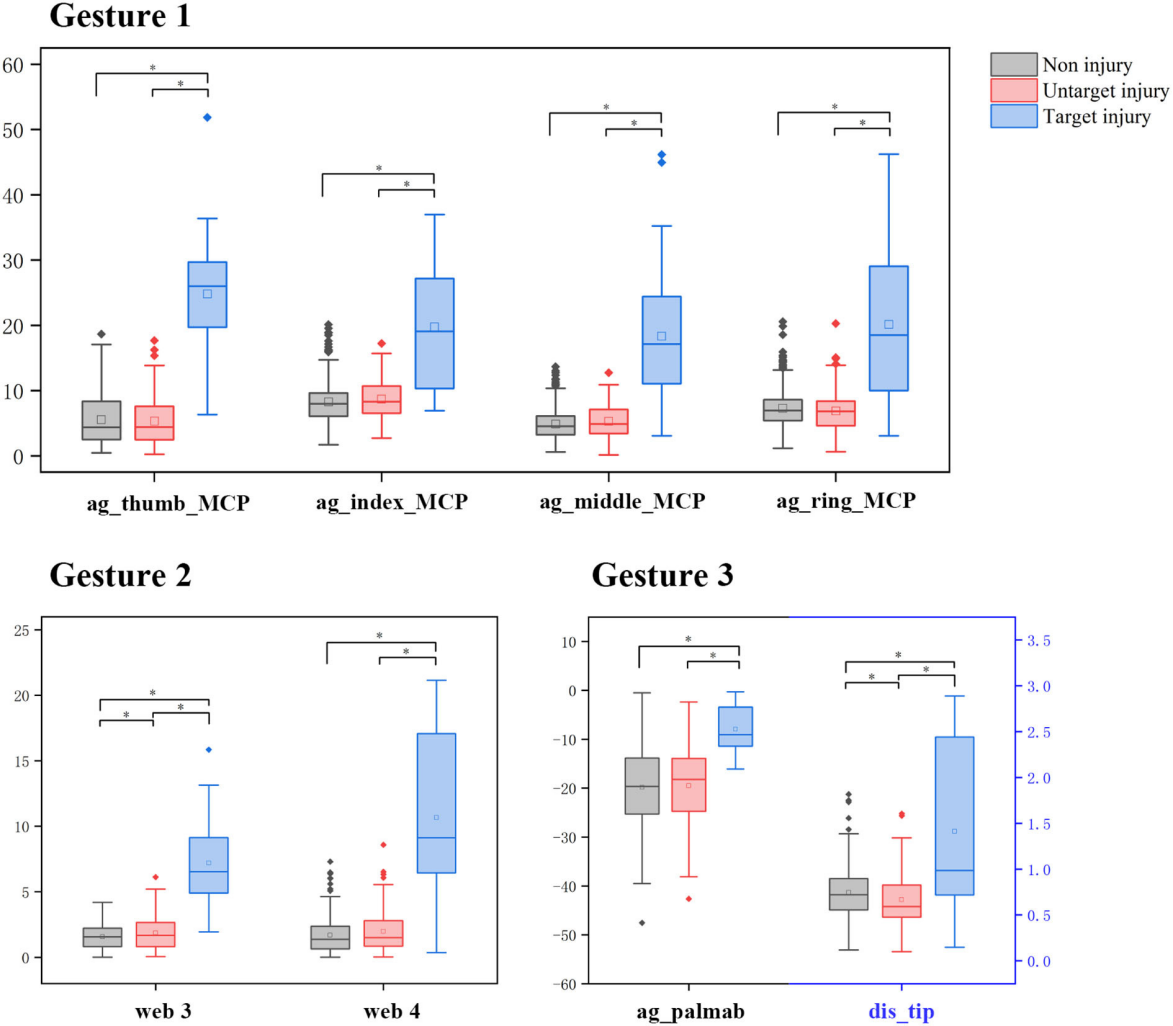
The subgroup analysis of features after selection. *Represents a significant difference among the hands with non-injury, untargeted injury, or targeted injury of all selected features (*P* < 0.05, independent *t*-test).

The statistical analysis of the predetermined features is shown in Table 1. In the independent *t*-test, all 23 predetermined features significantly differed between target and non-target groups, except 5 in Gesture 3. In the rule-based methods, eight features were selected after feature selection. In the subgroup analysis (shown in Figure 4), most selected features showed no significant difference in the non-target groups, except the angle of the third webspace and the tip distance between the thumb and the index finger. When compared with the ML method, selected features in the rule-based method also showed higher weight or importance in the ML models. For example, in Gesture 2, the angles of the third and fourth webspaces were selected in the rule-based model and were the only features in the logistic regression model. The hyperparameters, coefficients, and feature importance of all ML models are summarized in Supplemental material 1.

**Figure 5:**
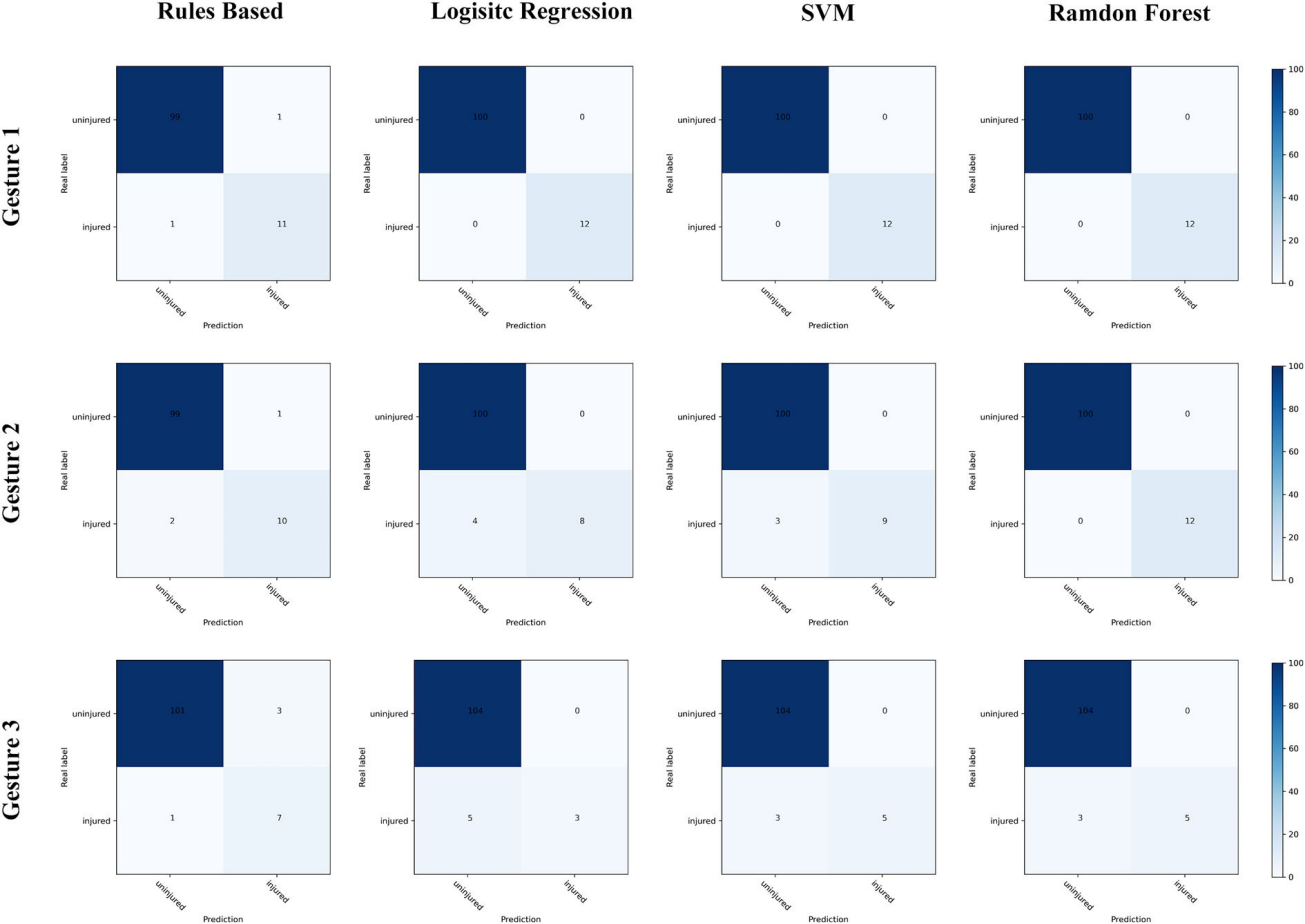
The performance of all classification models, shown in confusion matrixes.

The performance of all models is shown in Table 4, Figure 5. The accuracy, sensitivity, and specificity of rule-based models were (1) 98.2, 91.7, and 99.0% for radial nerve injury detection; (2) 97.3, 83.3% and 99.0% for ulnar nerve injury detection; and (3) 96.4, 87.5, and 97.1% for median nerve injury detection, respectively. In the ML models, radial nerve injury classification achieved the best performance with 100% accuracy. In all classification tasks, the rule-based method maintained sensitivity above 80%. In comparison, lower sensitivity appears in median nerve injury classification, with 62.5% in the SVM and RF models and only 37.5% in the RL model.

**Table 4:** Performance of all classification models.

	**Rules based**	**Logistic regression**	**SVM**	**Random forest**
**Gesture 1**
Accuracy	98.21%	100.00%	100.00%	100.00%
Sensitivity	91.67%	100.00%	100.00%	100.00%
Specificity	99.00%	100.00%	100.00%	100.00%
**Gesture 2**
Accuracy	97.32%	96.43%	97.32%	100.00%
Sensitivity	83.33%	66.67%	75.00%	100.00%
Specificity	99.00%	100.00%	100.00%	100.00%
**Gesture 3**
Accuracy	96.43%	95.54%	97.32%	97.32%
Sensitivity	87.50%	37.50%	62.50%	62.50%
Specificity	97.12%	100.00%	100.00%	100.00%

## Discussion

An abnormal gesture can be recognized by well-trained hand surgeons and indicates the presence of radial, ulnar, or median nerve injury before the diagnostic examination. In line with this diagnosis, we designed an automatic detection procedure for abnormal gestures enabled by an advanced hand pose estimation algorithm and rule-based models. According to the result, several features help detect the abnormalities from images to predict the type of nerve injuries. Nevertheless, our study is not intended to replace the role of clinicians or create a diagnostic method. We aimed to provide a simple, effective, and convenient way to alert the possible nerve injuries for unspecialized healthcare providers based on anatomical knowledge and clinical experience.

In this study, MediaPipe Hands, one of the novel pose estimation techniques, has been used as an automatic feature extraction method for classification tasks. We observed that the calculated angles from the landmark coordinates were not precisely in agreement with the manual assessment. For example, in the non-target groups of Gesture 1 and Gesture 2, all MCP joints and the PIP and DIP joints of the ring and the little fingers were higher than 5°. The mean value of the little MCP joint even reached 13.75°. When the digits are completely extended, these values are expected to be near 0° for people without nerve injury. There are several possible reasons to explain the differences. First, the pose estimation predicts the spatial coordinates of the landmark, yet manual goniometry usually measures the landmark on the surface of the hand. Second, the landmarks are not precisely the same, for there are no landmarks at the base of the metacarpals but only a single wrist landmark in MediaPipe skeleton models (24, 30), which may lead to noticeable differences in measuring the angles of MCP joints. In similar studies, a portable infrared camera called Leap Motion has been reported in functional assessments of hand rehabilitation (22). Researchers also found that the measurement result of Leap Motion was not favorable with manual measurement (31–34).

Under such circumstances, the threshold value of features could not be directly determined by clinical experience. Therefore, the training sets were used for feature selection and finding the cutoff. In the rule-based method, we used a filter strategy with a ROC curve because absolute distinctions exist between the injured and uninjured hands both in images and from the clinical practice experience. According to the independent *t*-test, all selected features could effectively categorize normal and abnormal gestures. Besides, the subgroup analysis further proved that, in each gesture, features between targeted and untargeted injuries were completely different, while features between untargeted injuries and non-injury were similar. In other words, our method is consistent with anatomical knowledge. Our study shows that MediaPipe Hands is competent at providing features for qualitative analysis. However, four unrecognized images in Gesture 3, one from the uninjured hand of a patient and three from the hand with radial nerve injury, also suggest that there is still room to propose estimation improvement. More annotated data and datasets are needed (35), especially for the medical population and the medical setting.

The rule-based models have been compared with three machine-learning models commonly used for binary classification in medical research (36–38). In the feature selection process, similar results are obtained for rule-based and ML methods, for the selected features in rule-based models have higher coefficients in the LR models and higher importance in the RF models. In the performance analysis, high specificity in all models shows the prediction for uninjured people is easy and correct. The sensitivity of ML models fluctuates from 37.5 to 100%. In comparison, the sensitivity of our models is stable from 83.3 to 91.7%, mainly because our rules seek the most essential and valuable features but might miss some synergistic effects among them. The overall performance of our methods is satisfactory. We believe that medical knowledge and clinical experience were the keys to maximizing the classification performance, even using minimal features and simple rules. They work as a shortcut to find the right features and make the detection performance in our tasks comparable to machine learning models.

The rule-based method is also interpretable (39, 40), which means the process is easier to modify and understand in human terms. It can provide expertise more conveniently and spread expert knowledge to primary healthcare providers. Tang et al. (11) reported a quantitative assessment method for upper-limb traumatic PNI. In their study, the presence of radial, ulnar, or median nerve injury was identified under an expert system using the data of surface EMG with 81.82% sensitivity and 98.90% specificity. Compared with Tang et al., our study possesses a little higher sensitivity and more straightforward implementation. Unlike surface EMG, taking images is more convenient and comfortable for patients. We are confident that our method has reliable results and is much easier to be applied by primary healthcare providers. As smartphones have become available and acceptable tools for telemedicine (41) and during the COVID-19 pandemic, performing virtual hand examinations through images and videos raises excellent interests in the medical field (42, 43). The proposed method has the potential to be a convenient screening tool for online health services and remote areas.

The relatively small sample size should be the main limitation of the present study. Since this was a prospective study and there was no available dataset of hand images with PNIs, continually updating our dataset is necessary to provide better automatic healthcare solutions. The smallest sample size in our study is patients with median nerve injury, which might weaken the generalizability of the proposed method. Although the differences between normal and abnormal gestures are noticeable, minor feature changes are highly possible when the sample size becomes more extensive. At present, our study only focuses on detecting nerve injuries and cannot detect abnormal gestures caused by other injuries, such as tendon rupture. In addition, the detection accuracy relies on the compliance of the users. Further study should invite more users in remote or rural areas to verify whether the rightful gestures can also be performed without the supervision of clinicians. Finally, our method functions as decision support to prevent missing nerve injuries in the primary assessment. After the initial discovery of the nerve injury, comprehensive examinations, differential diagnoses, and medical treatments are necessary.

## Data availability statement

The raw data supporting the conclusions of this article will be made available by the authors, without undue reservation.

## Ethics statement

The studies involving human participants were reviewed and approved by Institutional Review Board of The First Affiliated Hospital of Sun Yat-sen University (ID: [2021]387). Written informed consent for participation was not required for this study in accordance with the national legislation and the institutional requirements.

## Author contributions

XL, JY, and QZ contributed to the conception and design of the study. FG, JF, and CC performed the investigation and wrote the original draft. ZW organized the database and performed the statistical analysis. All authors contributed to the manuscript revision, read, and approved the submitted version.
